# Supplementary material for: The Drosophila proventriculus lacks stem cells but compensates for age-related cell loss via endoreplication-mediated cell growth
Source: Nat Commun. 2026 Jan 27;17:2086. doi: 10.1038/s41467-026-68876-5 (PMC12954101; doi:10.1038/s41467-026-68876-5)
Supplement: Supplementary file 1 — Supplementary Information [file 41467_2026_68876_MOESM1_ESM.pdf]

Supplementary Information:

The *Drosophila* proventriculus lacks stem cells but compensates for age-related cell loss via endoreplication-mediated cell growth

Supplementary Figures:

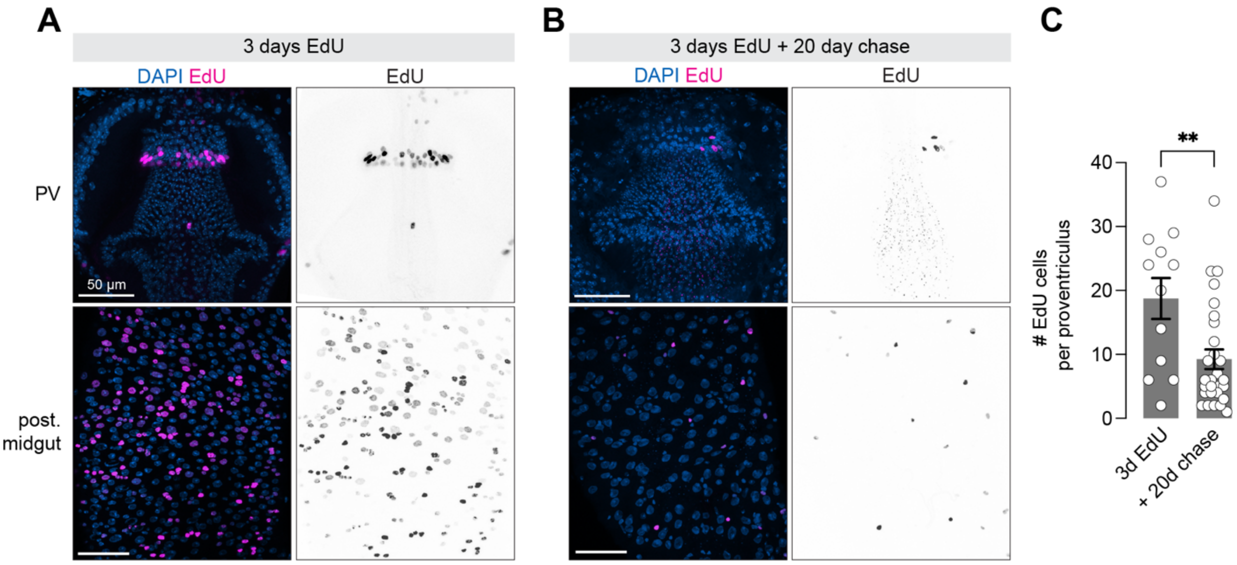

Supplementary Figure 1. EdU+ cells in Zone 4 cycle continuously. (A) After 3 days of EdU feeding, a ring of labeled cells is visible at the posterior edge of Zone 4, as well as in the ISCs and enterocytes of the midgut. (B) Following a 20 day chase period, a reduction of EdU+ cells is visible in both Zone 4 cells and in the midgut, where continual cycling has diluted out the original EdU pulse. (C) Quantification of data shown in A-B. N = 3d: 9 independent proventriculi, 20d chase: 22 independent proventriculi. Statistical test is an unpaired t-test; ** = 0.0024. “PV” = proventriculus. “post-midgut” = posterior midgut.

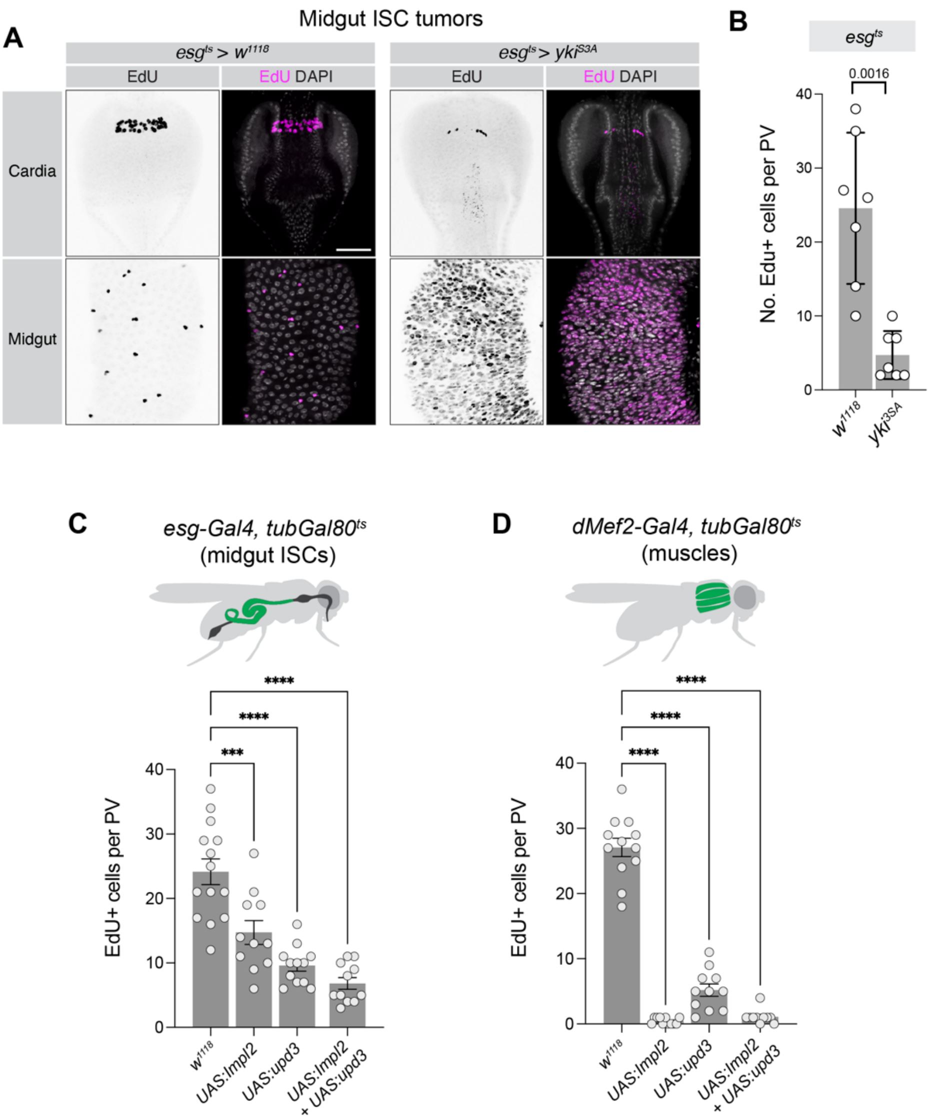

Supplementary Figure 2. Systemic reduction in insulin signaling reduces the number of EdU+ cells in the proventriculus. (A) Tumors in midgut ISCs (driven by *esg-Gal4*^{ts} > *yki*^{3SA}) cause a reduction in EdU+ cells in the proventriculus. Note that *esg-Gal4* is not expressed in the proventriculus. Anterior is up, scale bar is 50 μm. (B) Quantification of the experiment shown in (A). N = 7 independent proventriculi. Statistical test is Welch's t-test. (C) Over-expression of insulin-antagonists *ImpL2, upd3,* or a combination of both from the ISCs (*esg-Gal4*) leads to a reduction in EdU+ cells. N = independent proventriculi: *w*^{1118} = 14; *UAS:Impl2* = 11; *UAS:upd3* = 12; *UAS:Impl2+UAS:upd3* = 11. Statistical tests are one-way ANOVA; *** p = 0.0003; **** p < 0.0001. (D) Over-expression of insulin-antagonists *ImpL2, upd3,* or a combination of both from muscles using *dMef2-Gal4* leads to a reduction in EdU+ cells. N = independent proventriculi: *w*^{1118} = 12; *UAS:Impl2* = 11; *UAS:upd3* = 11; *UAS:Impl2+UAS:upd3* = 10. Statistical tests are one-way ANOVA; **** p < 0.0001.

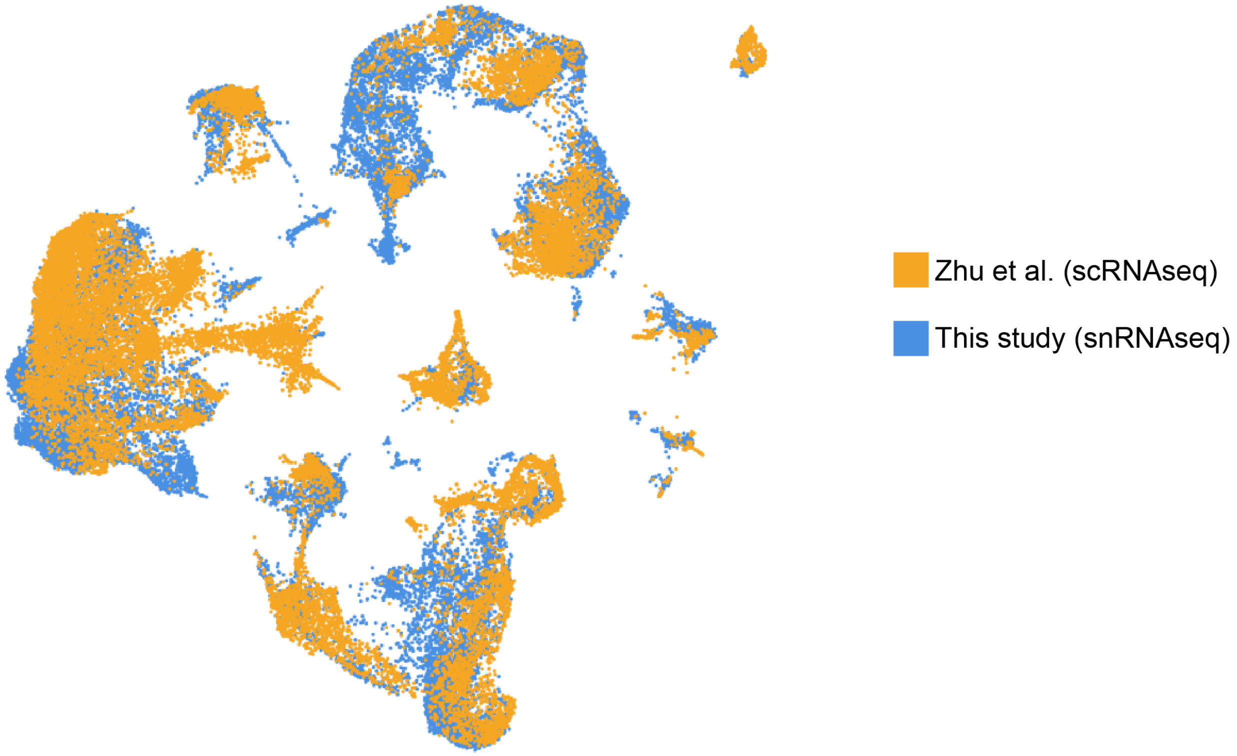

Supplementary Figure 3. Merged UMAP projection of two single cell atlases of the proventriculus. Data from the snRNAseq atlas presented in this manuscript and scRNAseq data from Zhu *et al.* (2024) [main referenance 25] were bioinformatically pooled and re-analyzed into a single dataset.

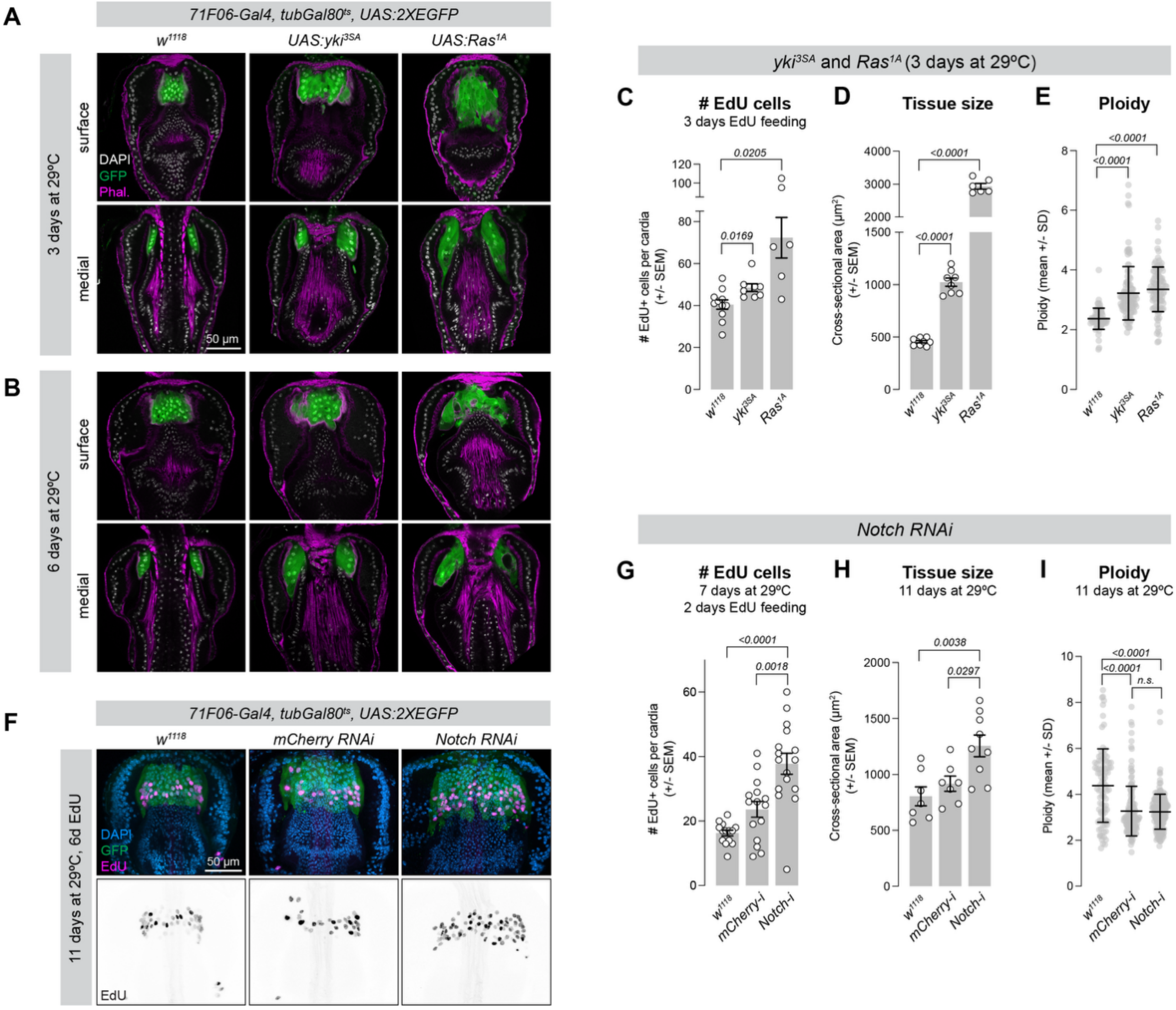

Supplementary Figure 4. Additional characterization of *yki^{3SA}*, *Ras^{1A}*, and *Notch-RNAi* phenotypes in proventriculus Zone 4 cells. (A-B) Ectopic expression of oncogenes *yki^{3SA}* or *Ras^{1A}* in Zone 4 cells of the proventriculus for either (A) three days or (B) six days leads to tissue enlargement, yet no GFP+ cells are visible outside of Zone 4. (C) *71F06-Gal4^{ts}* > *yki^{3SA}* or *Ras^{1A}* leads to increased number of EdU+ cells. N = independent proventriculi: *w^{1118}* = 11; *yki^{3SA}* = 8; *Ras^{1A}* = 6. Statistical test is two-tailed unpaired t-test. (D) *71F06-Gal4^{ts}* > *yki^{3SA}* or *Ras^{1A}* leads to increased tissue size, measured as cross-sectional area at the tissue midpoint. Each value represents the average of two measures per proventriculus, on either side of the esophagus. N = independent proventriculi: *w^{1118}* = 9; *yki^{3SA}* = 8; *Ras^{1A}* = 6. Statistical test is two-one-way ANOVA with Tukey's multiple comparison test (E) *71F06-Gal4^{ts}* > *yki^{3SA}* or *Ras^{1A}* leads to increased ploidy. Each point represents a single nucleus; N = number of nuclei: *w^{1118}* = 76; *yki^{3SA}* = 110; *Ras^{1A}* = 98, measured from 4 independent proventriculi. (F) *71F06-Gal4^{ts}* > *Notch-RNAi* leads to increased tissue size and increased number of EdU cells. (G) Number of EdU+ cells in *71F06-Gal4^{ts}* > *Notch-RNAi* flies is significantly increased compared to controls. N = independent proventriculi: *w^{1118}* = 14; *mCherry-RNAi* = 15; *Notch-RNAi* = 16. Statistical test is one-way ANOVA with Tukey's multiple comparison test. (H) *71F06-Gal4^{ts}* > *Notch-RNAi* causes increased Zone 4 tissue size. N = independent proventriculi: *w^{1118}* = 7; *mCherry-RNAi* = 7; *Notch-RNAi* = 9. Statistical test is one-way ANOVA with Tukey's multiple comparison test. (I) Ploidy is not

increased in *71F06-Gal4*^{ts} > *Notch-RNAi,* and does not significantly differ from *mCherry-RNAi* controls. N = number of nuclei: *w*^{1118} = 144; *mCherry-RNAi* = 92; *Notch-RNAi* = 201. N = number of independent proventriculi: *w*^{1118} = 5; *mCherry-RNAi* = 7; *Notch-RNAi* = 7. Statistical test is one-way ANOVA with Tukey's multiple comparison test.

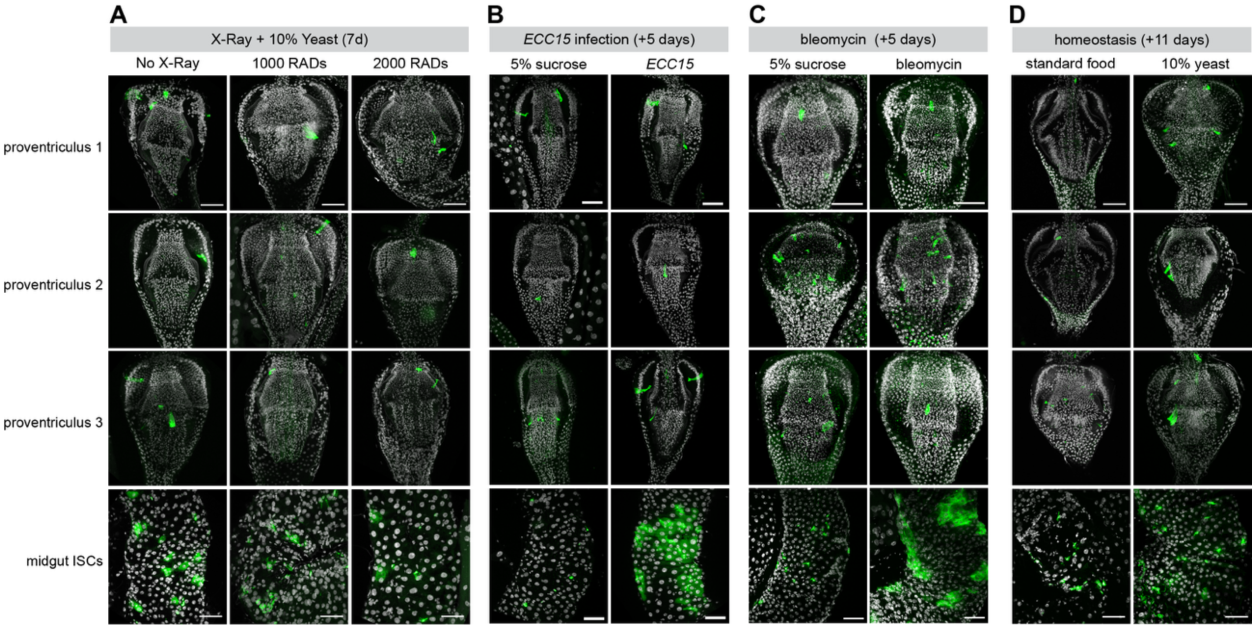

Supplementary Figure 5. MARCM analysis in the proventriculus following gut damage. Analysis of MARCM clones in the proventriculus and midgut following a number of perturbations to the gut. Three representative proventriculus images are shown for each condition, from a total sample size between 10 and 23 guts. (A) X-ray damage to entire flies at 1000 or 2000 RADS, examined seven days after damage. (B) Gut damage from *ECC15* bacteria, examined 5 days after damage. (C) Gut damage from bleomycin, examined 11 days after damage. (D) Undamaged guts, fed with food supplemented with 10% yeast. Anterior is up, scale bar is 50µm.

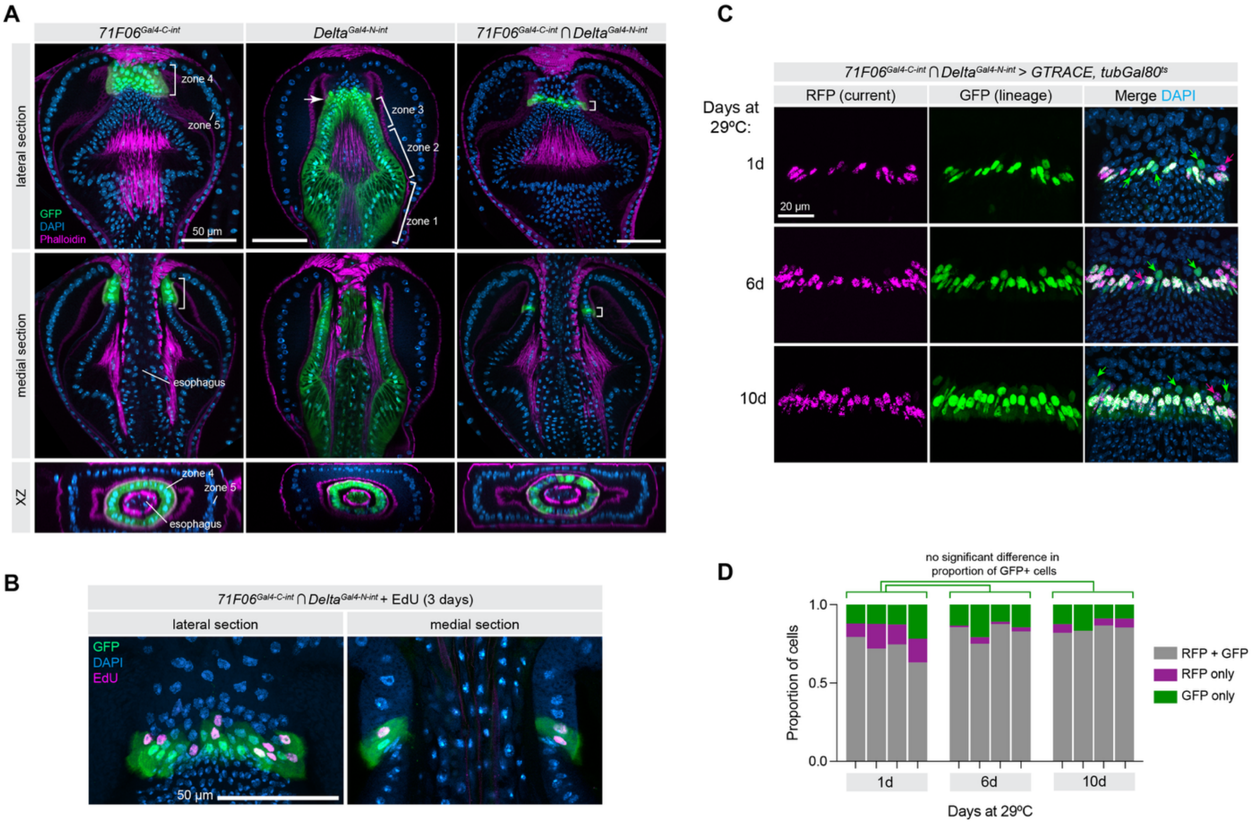

Supplementary Figure 6. Genetic lineage tracing using a highly specific split-intein Gal4 that labels Edu+ cells. (A) Expression pattern of *71F06*$^{Gal4\text{-}C\text{-}int}$, *Delta*$^{Gal4\text{-}N\text{-}int}$, and the intersection of *71F06 ∩ Delta*. (B) *71F06 ∩ Delta* expression largely overlaps with EdU+ cells in Zone 4 cells. (C) Using *71F06 ∩ Delta* to drive the G-TRACE lineage system, with *tubGal80*^{ts} to restrict labeling to the adult stage for the indicated duration. Colored arrows indicate individual cells with either solely GFP (past but not current expression) or RFP (very recently initiated expression.) Anterior is up. (D) Quantification of experiment shown in (C). Each column represents a single proventriculus sample at the indicated time-point. Statistical comparison is calculated as ordinary one-way ANOVA comparing the proportion of GFP-only cells between time-points. N = 4 independent proventriculi at each time point. Statistical test is Kruskal Wallace test, p = 0.5101.

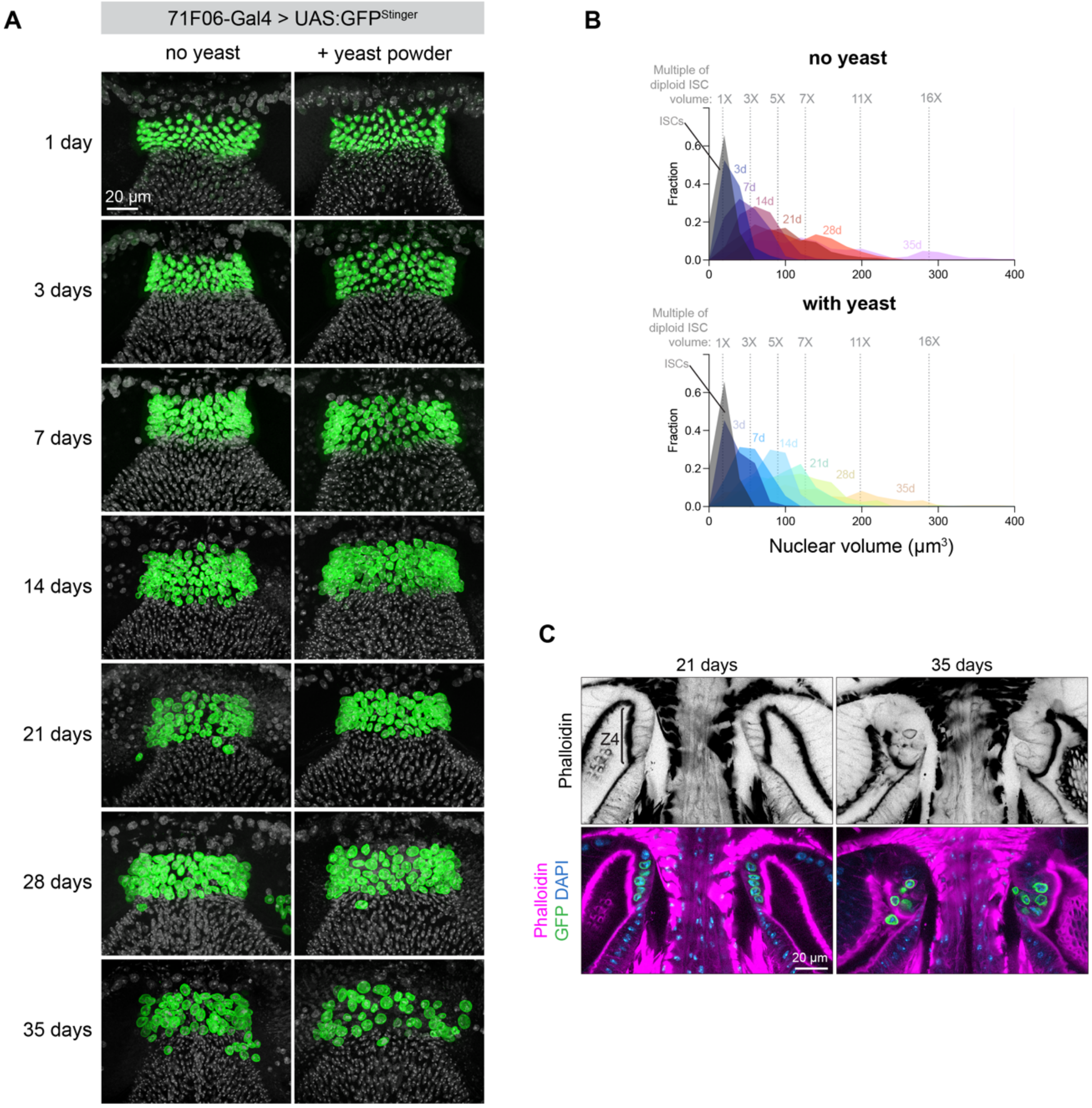

Supplementary Figure 7. Time course analysis of nuclear volume and morphology in Zone 4 cells. (A) Representative images of each time-point for the experiment shown in Figure 7. (B) Histogram of nuclear volume calculated from the data represented in Figure 7. (C) 71F06> Stinger flies aged 21d (left) or 35d (right) and stained for phalloidin, indicating that cells remain mononucleate throughout this time-course. Z4 = zone 4. Anterior is up.

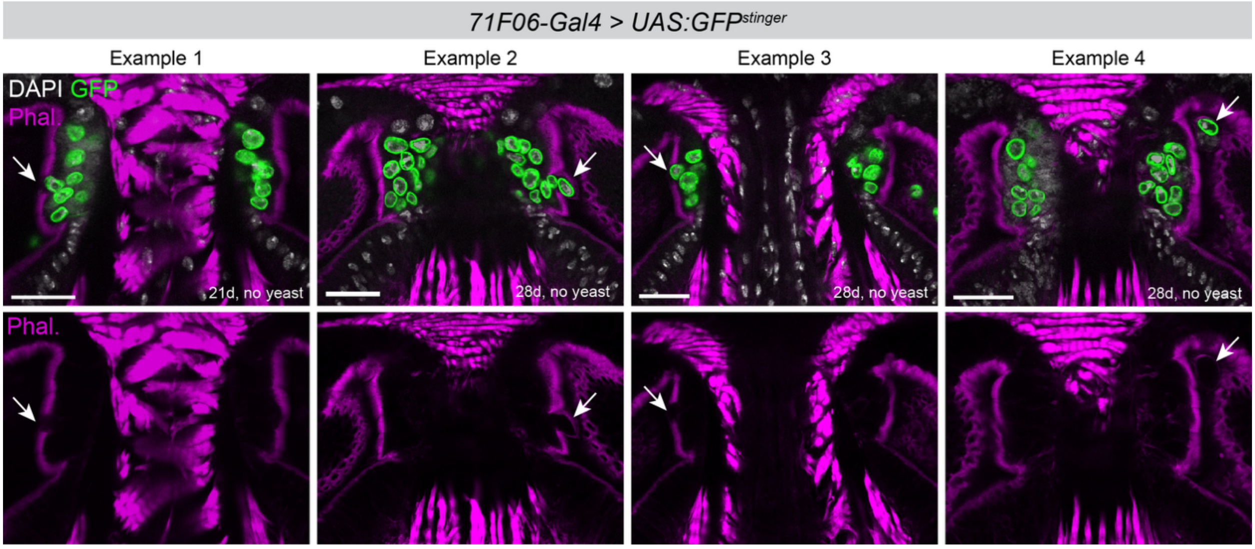

Supplementary Figure 8. Additional examples of Zone 4 nuclei loss into the gut lumen. In aging flies, GFP+ nuclei can be seen shed into the gut lumen from Zone 4. Multiple examples are shown of these nuclei appearing to exit Zone 4 through disruptions in the apical (lumen-facing) surface of Zone 4, visible as loss of phalloidin. Cells are observed at various locations along the anterior-posterior axis of Zone 4. Anterior is up, scale bar is 20μm.

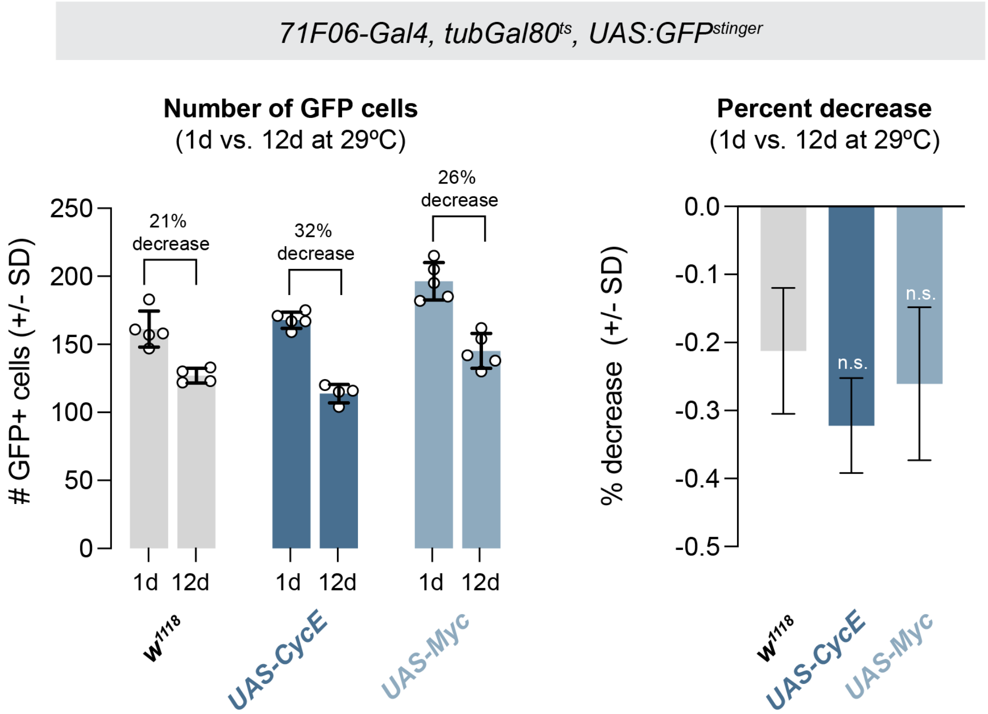

Supplementary Figure 9. Increased endocycling does not enhance cell loss in Zone 4. Left: Cell counts of GFP+ Zone 4 cells after 1 day or 12 days at 29ºC for the indicated genotypes. N = independent proventriculi (1d, 12); w^{1118} = 5, 4; *CycE* = 5, 4; *Myc* = 5,5. Right: the average percentage decrease, calculated from the data shown on the left, was not significantly different between these conditions. Error bars represent standard deviation propagated through the calculation of percentage decrease, and mean differences were compared using a two-way unpaired t-test.

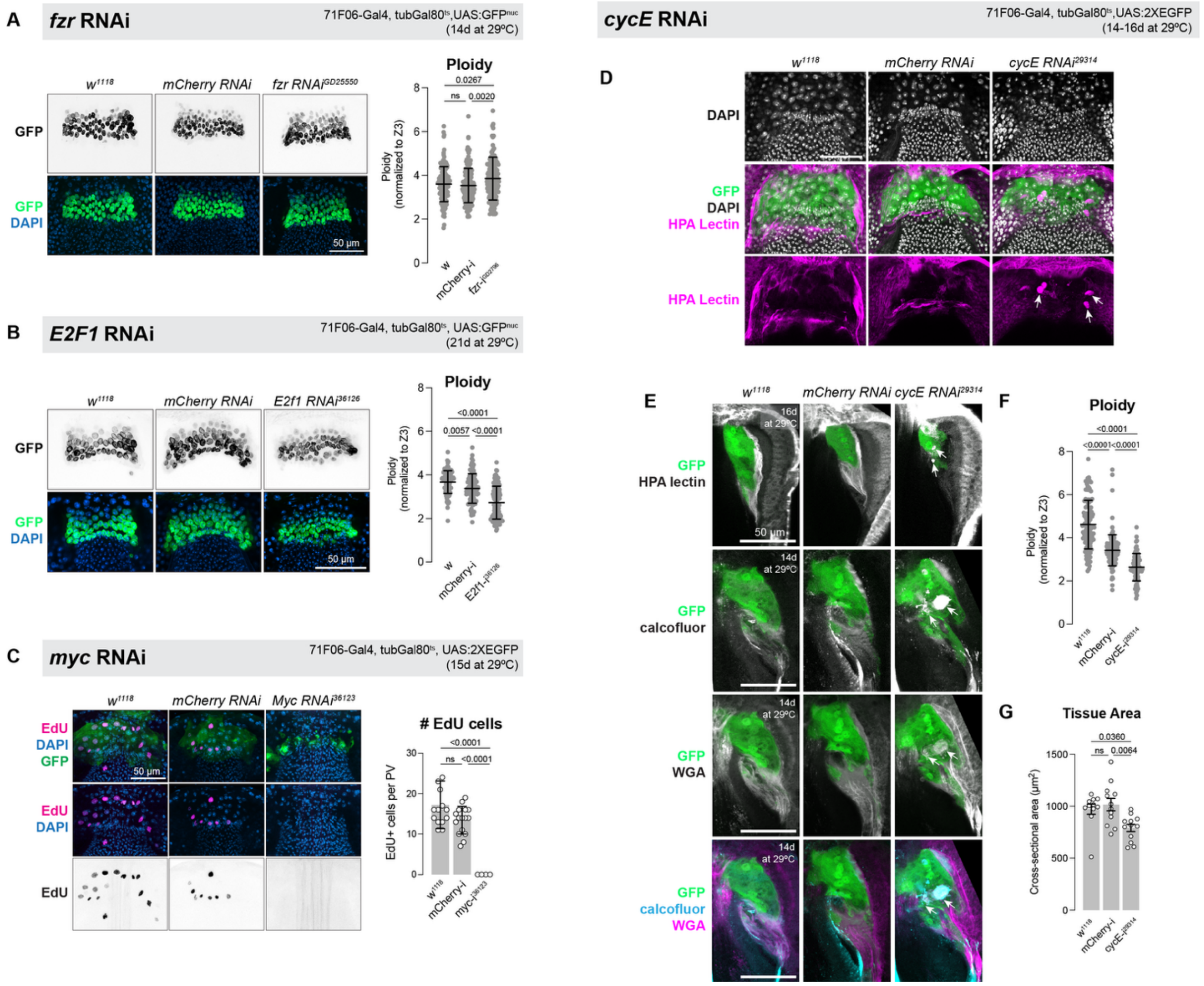

Supplementary Figure 10. RNAi against additional candidates implicated in endocycling. (A) RNAi against *fzr* does not reduce ploidy in Zone 4, and in fact slightly increases cell ploidy. Representative images are shown to the left, quantification of ploidy shown at right. N = number of nuclei: *w* = 153; *mCherry-RNAi* = 180*; fzr-RNAi* = 160. N = number of independent proventriculi = 5. Statistical test is one-way ANOVA with Tukey's multiple test correction. (B) RNAi against *E2F1* significantly reduces ploidy in Zone 4, to a lesser degree than *InR-RNAi.* Representative images are shown to the left, quantification of ploidy shown at right. N = number of nuclei: *w* = 105; *mCherry-RNAi* = 99*; fzr-RNAi* = 124. N = number of independent proventriculi; *w* = 5; *mCherry-RNAi* = 4; *E2F1-RNAi* = 5. Statistical test is one-way ANOVA with Tukey's multiple test correction. (C) RNAi against *myc* abolishes EdU incorporation (right) but also massively disrupts or destroys Zone 4 morphology (left.) (D) Representative images of *71F06-Gal4*^{ts} > *cycE-RNAi,* demonstrating smaller nuclei (top row) and aggregates of peritrophic membrane, revealed via HPA lectin staining (bottom row, arrows.) (E) RNAi against *cycE* causes aggregates of peritrophic membrane in Zone 4 that stains for HPA lectin, calcofluor, and WGA (arrows). (F) *71F06-Gal4*^{ts} > *cycE-RNAi* reduces ploidy. N = number of nuclei: *w* = 110; *mCherry-RNAi* = 136*; fzr-RNAi* = 79. N = number of independent proventriculi; *w* = 5; *mCherry-RNAi* = 5; *cycE-RNAi* = 4. Statistical test is one-way ANOVA with Tukey's multiple test correction. (G)

71F06-Gal4^{ts} > *cycE-RNAi* reduces tissue size compared to controls. Each point represents the average of two measurements per proventriculus. N = number of independent proventriculi: *w^{1118}* = 11, *mCherry-RNAi* = 12, *cycE-RNAi* = 12. Statistical test is one-way ANOVA with Tukey's multiple test correction.

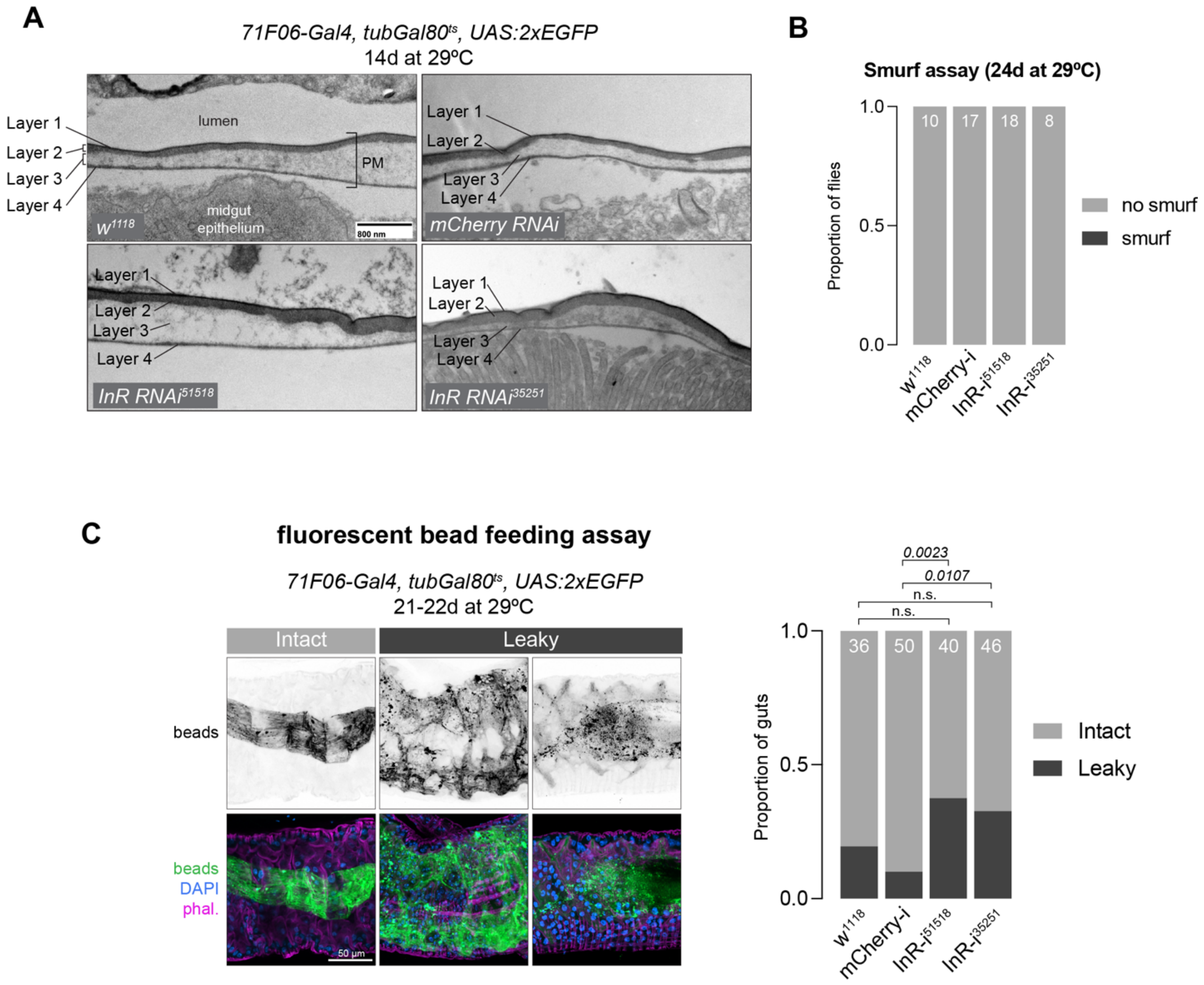

Supplementary Figure 11. Additional characterization of peritrophic membrane in *71F06^{ts} > InR-RNAi flies.* (A) Representative transmission electron microscopy images of peritrophic membrane in the anterior midgut of the indicated genotypes. The four-layered structure of the PM is visible in each case, with no gross morphological defects in *InR-RNAi* flies. (B) "Smurf" assay for leaky guts did not reveal any gut-defective flies after 24 days of RNAi expression. (C) Fluorescent bead retention assay. (Left) Examples of intact versus leaky guts that each sample was scored for. In intact guts, fluorescent beads were restricted to a "sleeve" of peritrophic membrane, whereas in "leaky" guts beads could be seen extending out to contact the gut epithelium. (Right) Quantification of leaky guts for each genotype. Three separate experiments were pooled, with total sample size given at the top of each column. Statistical tests are two-sided Fisher's Exact Test.

Supplementary Table 1: Genotypes used in this study

Figure	Genotype	Source Notes
1B, left	*yw ; + / wg[Sp-1] ; TI{GFP[3xP3.cLa]=CRIMIC.TG4.0}Chs2[CR60212-TG4.0] / P{w[+mC]=UAS-2xEGFP}AH3*	Chs2-Gal4: BL93534; UAS:2xEGFP: BL60293
1B, middle	*w ; P{w[+mC]=UAS-2xEGFP}AH2 ; P{w[+mW.hs]=GawB}Lk6[NP0101] / P{y[+t7.7] v[+t1.8]=VALIUM20-mCherry.RNAi}attP2*	"Cardia-Gal4": Kyoto DGRC 103522; UAS:2xEGFP: BL60292; mCherry RNAi: BL35785
1B, right	*w[1118] ; esg-Gal4, UAS:GFP, tubGal80[ts]*	Perrimon lab stock
1C, left	*w[1118] ; esg-Gal4, UAS:GFP, tubGal80[ts]*	Perrimon lab stock
1C right	*w[1118]; P{w[+mC]=10XStat92E-GFP}1*	Perrimon lab stock
1D	*Oregon R*	
1E	*Oregon R*	
1F,G	*w[1118] ; esg-Gal4, UAS:GFP, tubGal80[ts]*	Perrimon lab stock
2A	*w ; P{UAS-Stinger}2 / + ; P{y[+t7.7] w[+mC]=GMR71F06-GAL4}attP2 / MKRS*	UAS-Stinger: BL90920, GMR71F06-Gal4: BL39596
2E	*yw ; wg[Sp-1] / + ; Mi{Trojan-GAL4.0}Elovl7[MI01455-TG4.0] / P{w[+mC]=UAS-2xEGFP}AH3*	Elovl7-Gal4: BL67431
2E	*yw ; wg[Sp-1] / + ; TI{GFP[3xP3.cLa]=CRIMIC.TG4.2}CG11550[CR02741-TG4.2] / P{w[+mC]=UAS-2xEGFP}AH3*	CG11550-Gal4: BL97589
2E	*yw ; P{y[+t7.7] w[+mC]=Tub-GAL4.C-int}attP40 / + ; TI{RFP[3xP3.PB]=2A-GAL4(1-20)::N-int}Ppn[G4-N-int] / P{w[+mC]=UAS-2xEGFP}AH3*	Ppn-C-int: BL602730; tub-Gal4-C-int: BL602738
2E	*yw ; wg[Sp-1] / + ; TI{GFP[3xP3.cLa]=CRIMIC.TG4.2}Lgr1[CR01685-TG4.2] / P{w[+mC]=UAS-2xEGFP}AH3*	Lgr1-Gal4: BL86491
2E	*yw ; y[1] w[*]; Mi{Trojan-GAL4.2}Wnt4[MI03717-TG4.2] / wg[Sp-1] ; P{w[+mC]=UAS-2xEGFP}AH3*	Wnt4-Gal4: BL67449
2E	*yw ; TI{GFP[3xP3.cLa]=CRIMIC.TG4.2}Ca-Ma2d[CR01742-TG4.2] / wg[Sp-1] ; P{w[+mC]=UAS-2xEGFP}AH3*	Ca-Ma2d-Gal4: BL86499
2E	*w[*] TI{RFP[3xP3.PB]=2A-GAL4(1-20)::N-int}Mco4[G4-N-int] ; P{y[+t7.7] w[+mC]=Tub-GAL4.C-int}attP40 / + ; P{w[+mC]=UAS-2xEGFP}AH3 / +*	Mco4-N-int: BL605167; tub-Gal4-C-int: BL602738
2E	*yw ; wg[Sp-1] / + ; TI{GFP[3xP3.cLa]=CRIMIC.TG4.2}SecCl[CR01836-TG4.2]/TM3, Sb[1] Ser[1] / P{w[+mC]=UAS-2xEGFP}AH3*	SecCl-Gal4: BL91420
2E	*w ; Mex1-Gal4, UAS-2xEGFP*	Mex1-Gal4 gift of A. Petsakou (originally from Phillips & Thomas, 2006)
3A, left	*w ; P{w[+mC]=tubP-GAL80[ts]}10], P{w[+mC]=UAS-2xEGFP}AH2 ; P{y[+t7.7] w[+mC]=GMR71F06-GAL4}attP2*	GMR71F06-Gal4: BL39596, UAS:2xEGFP:

		BL60292, tubGal80ts: BL7108
3A, second to left	*w ; P{w[+mC]=tubP-GAL80[ts]}10], P{w[+mC]=UAS-2xEGFP}AH2 ; P{y[+t7.7] w[+mC]=GMR71F06-GAL4}attP2 / P{y[+t7.7] w[+mC]=UAS-yki.S111A.S168A.S250A.V5}attP2*	yki[3SA]: BL28817
3A, third to left	*w ; P{w[+mC]=tubP-GAL80[ts]}10], P{w[+mC]=UAS-2xEGFP}AH2 ; P{y[+t7.7] w[+mC]=GMR71F06-GAL4}attP2 / UAS:Ras[1A]*	Ras1A: gift of Chiwei Xu, Perrimon Lab
3A, fourth to left	*w ; P{w[+mC]=tubP-GAL80[ts]}10], P{w[+mC]=UAS-2xEGFP}AH2 ; P{y[+t7.7] w[+mC]=GMR71F06-GAL4}attP2*	
3A, right	*w ; P{w[+mC]=tubP-GAL80[ts]}10], P{w[+mC]=UAS-2xEGFP}AH2 ; P{y[+t7.7] w[+mC]=GMR71F06-GAL4}attP2 / P{y[+t7.7] v[+t1.8]=TRiP.HMS00001}attP2*	Notch-RNAi: BL33611
4	hsFLP, FRT19A, tubGal80 / P{ry[+t7.2]=neoFRT}19A; ry[506] ; ; tub-Gal4, UAS-GFP	FRT19A: BL1709; MARCM19A: Gift of P. Jouandin
5A, top	*w ; P{w[+mC]=UAS-rpr.C}14 / + ; P{w[+mC]=tubP-GAL80[ts]}ncd[GAL80ts-7] / +*	UAS-rpr: BL5824; tubGal80ts: BL7018
5A, bottom	*w ; P{w[+mC]=UAS-rpr.C}14 / TI{GFP[3xP3.cLa]=CRIMIC.TG4.2}Ca-Ma2d[CR01742-TG4.2] ; P{w[+mC]=tubP-GAL80[ts]}ncd[GAL80ts-7] / +*	Elovl7-Gal4: BL67431
5B, top	*w ; P{w[+mC]=UAS-rpr.C}14 / + ; P{w[+mC]=tubP-GAL80[ts]}ncd[GAL80ts-7] / +*	
5B, bottom	*w ; P{w[+mC]=UAS-rpr.C}14 / + ; P{w[+mC]=tubP-GAL80[ts]}ncd[GAL80ts-7] / TI{GFP[3xP3.cLa]=CRIMIC.TG4.2}CG11550[CR02741-TG4.2]*	CG11550-Gal4: BL97589
5C, top	*w ; P{w[+mC]=UAS-rpr.C}14 / + ; P{w[+mC]=tubP-GAL80[ts]}ncd[GAL80ts-7] / +*	
5C, bottom	*w ; P{w[+mC]=UAS-rpr.C}14 / + ; P{w[+mC]=tubP-GAL80[ts]}ncd[GAL80ts-7] / Mi{Trojan-GAL4.0}Elovl7[MI01455-TG4.0]*	Ca-Ma2d-Gal4: BL86499
6	*w ; P{UAS-Stinger}2 / + ; P{y[+t7.7] w[+mC]=GMR71F06-GAL4}attP2 / MKRS*	UAS-Stinger: BL90920, GMR71F06-Gal4: BL39596
7B, left	*w ; P{w[+mC]=UAS-rpr.C}14 / + ; P{w[+mC]=tubP-GAL80[ts]}ncd[GAL80ts-7] / +*	UAS-rpr: BL5824; tubGal80ts: BL7018
7B, right	*w ; P{w[+mC]=UAS-rpr.C}14 / + ; P{w[+mC]=tubP-GAL80[ts]}ncd[GAL80ts-7] / P{y[+t7.7] w[+mC]=GMR71F06-GAL4}attP2*	GMR71F06-Gal4: BL39596
7E	*OregonR*	
8A, left	*w ; P{w[+mC]=tubP-GAL80[ts]}10], P{w[+mC]=UAS-2xEGFP}AH2 ; P{y[+t7.7] w[+mC]=GMR71F06-GAL4}attP2 / +*	
8A, second to left	*w ; P{w[+mC]=tubP-GAL80[ts]}10], P{w[+mC]=UAS-2xEGFP}AH2 ; P{y[+t7.7] w[+mC]=GMR71F06-GAL4}attP2 / P{w[+mC]=UAS-CycE.L}ML1*	UAS-CycE: BL4781
8A, second to right	*w ; P{w[+mC]=tubP-GAL80[ts]}10], P{w[+mC]=UAS-2xEGFP}AH2 ; P{y[+t7.7] w[+mC]=GMR71F06-GAL4}attP2 / +*	

8A, right	*w ; P{w[+mC]=tubP-GAL80[ts]}10], P{w[+mC]=UAS-2xEGFP}AH2 ; P{y[+t7.7] w[+mC]=GMR71F06-GAL4}attP2 / M{w[+mC]=UAS-Myc.HA.WT}ZH-86Fb*	UAS-Myc: BL64759
8B, left	*w ; P{UAS-Stinger}2, P{w[+mC]=tubP-GAL80[ts]}10] / + ; P{y[+t7.7] w[+mC]=GMR71F06-GAL4}attP2 / +*	
8B, middle	*w ; P{UAS-Stinger}2, P{w[+mC]=tubP-GAL80[ts]}10] / + ; P{y[+t7.7] w[+mC]=GMR71F06-GAL4}attP2 / P{w[+mC]=UAS-CycE.L}ML1*	UAS-CycE: BL4781
8A, right	*w ; P{UAS-Stinger}2, P{w[+mC]=tubP-GAL80[ts]}10] / + ; P{y[+t7.7] w[+mC]=GMR71F06-GAL4}attP2 / M{w[+mC]=UAS-Myc.HA.WT}ZH-86Fb*	UAS-Myc: BL64759
8C, D, E left	*w ; P{w[+mC]=tubP-GAL80[ts]}10], P{w[+mC]=UAS-2xEGFP}AH2 ; P{y[+t7.7] w[+mC]=GMR71F06-GAL4}attP2 / +*	
8C, D, E middle	*w ; P{w[+mC]=tubP-GAL80[ts]}10], P{w[+mC]=UAS-2xEGFP}AH2 ; P{y[+t7.7] w[+mC]=GMR71F06-GAL4}attP2 / P{w[+mC]=UAS-CycE.L}ML1*	UAS-CycE: BL4781
8C, D, E right	*w ; P{w[+mC]=tubP-GAL80[ts]}10], P{w[+mC]=UAS-2xEGFP}AH2 ; P{y[+t7.7] w[+mC]=GMR71F06-GAL4}attP2 / M{w[+mC]=UAS-Myc.HA.WT}ZH-86Fb*	UAS-Myc: BL64759
9A, B	*w ; P{w[+mC]=tubP-GAL80[ts]}10], P{w[+mC]=UAS-2xEGFP}AH2 ; P{y[+t7.7] w[+mC]=GMR71F06-GAL4}attP2 / +*	
	w / y sc v sev; P{w[+mC]=tubP-GAL80[ts]}10], P{w[+mC]=UAS-2xEGFP}AH2 ; P{y[+t7.7] w[+mC]=GMR71F06-GAL4}attP2 / P{y[+t7.7] v[+t1.8]=VALIUM20-mCherry.RNAi}attP2	mCherry RNAi: BL35785
	w / y v ; P{w[+mC]=tubP-GAL80[ts]}10], P{w[+mC]=UAS-2xEGFP}AH2 / P{y[+t7.7] v[+t1.8]=TRiP.HMS03166}attP40 ; P{y[+t7.7] w[+mC]=GMR71F06-GAL4}attP2 / +	InR RNAi: BL51518
	w / y sc v sev; P{w[+mC]=tubP-GAL80[ts]}10], P{w[+mC]=UAS-2xEGFP}AH2 ; P{y[+t7.7] w[+mC]=GMR71F06-GAL4}attP2 / P{y[+t7.7] v[+t1.8]=TRiP.GL00139}attP2	InR RNAi: BL35251
9C	Same as 9A, except UAS-Stinger instead of UAS-2xEGFP	
9D-G	Same as 9A	
S1	*Oregon R*	
S2A, left	*w ; esg-Gal4, UAS:GFP, tubGal80[ts] ; +*	
S2A, right	*w ; esg-Gal4, UAS:GFP, tubGal80[ts] ; P{y[+t7.7] w[+mC]=UAS-yki.S111A.S168A.S250A.V5}attP2*	
S2C	*w ; esg-Gal4, UAS:GFP, tubGal80[ts] ; +*	
S2C	*w ; esg-Gal4, UAS:GFP, tubGal80[ts] ; UAS:Impl2*	UAS:ImpL2: Gift of Ying Liu
S2C	*w ; esg-Gal4, UAS:GFP, tubGal80[ts] / UAS:upd3 ; +*	UAS:upd3: Gift of Ying Liu
S2C	*w ; esg-Gal4, UAS:GFP, tubGal80[ts] / UAS:upd3 ; UAS:Impl2*	UAS:Impl2; UAS:upd3; Gift of A. Petsakou
S2D	*w ; dMef2-Gal4 / + ; tubGal80[ts] / +*	dMef2-Gal4: Perrimon Lab stock
S2D	*w ; dMef2-Gal4 / UAS:Impl2 ; tubGal80[ts] / +*	UAS:ImpL2: Gift of Ying Liu

S2D	*w ; dMef2-Gal4 / + ; tubGal80[ts] / UAS:upd3*	UAS:upd3: Gift of Ying Liu
S2D	*w ; dMef2-Gal4 / UAS:upd3 ; tubGal80[ts] / UAS:Impl2*	UAS:Impl2; UAS:upd3; Gift of A. Petsakou
S3	hsFLP, FRT19A, tubGal80 / P{ry[+t7.2]=neoFRT}19A; ry[506] ; ; tub-Gal4, UAS-GFP	FRT19A: BL7109; MARCM19A: Gift of P. Jouandin
S4A-E, left	*w ; P{w[+mC]=tubP-GAL80[ts]}10], P{w[+mC]=UAS-2xEGFP}AH2 ; P{y[+t7.7] w[+mC]=GMR71F06-GAL4}attP2*	GMR71F06-Gal4: BL39596, UAS:2xEGFP: BL60292, tubGal80ts: BL7108
S4A-E, middle	*w ; P{w[+mC]=tubP-GAL80[ts]}10], P{w[+mC]=UAS-2xEGFP}AH2 ; P{y[+t7.7] w[+mC]=GMR71F06-GAL4}attP2 / P{y[+t7.7] w[+mC]=UAS-yki.S111A.S168A.S250A.V5}attP2*	yki[3SA]: BL28817
S4A-E, right	*w ; P{w[+mC]=tubP-GAL80[ts]}10], P{w[+mC]=UAS-2xEGFP}AH2 ; P{y[+t7.7] w[+mC]=GMR71F06-GAL4}attP2 / Ras[1A]*	Ras1A: gift of Chiwei Xu, Perrimon Lab
S4F-I, left	*w ; P{w[+mC]=tubP-GAL80[ts]}10], P{w[+mC]=UAS-2xEGFP}AH2 ; P{y[+t7.7] w[+mC]=GMR71F06-GAL4}attP2*	
S4F-I, middle	*w ; P{w[+mC]=tubP-GAL80[ts]}10], P{w[+mC]=UAS-2xEGFP}AH2 ; P{y[+t7.7] w[+mC]=GMR71F06-GAL4}attP2 / P{y[+t7.7] v[+t1.8]=VALIUM20-mCherry.RNAi}attP2*	mCherry RNAi: BL35785
S4F-I, right	*w ; P{w[+mC]=tubP-GAL80[ts]}10], P{w[+mC]=UAS-2xEGFP}AH2 ; P{y[+t7.7] w[+mC]=GMR71F06-GAL4}attP2 / P{y[+t7.7] v[+t1.8]=TRiP.HMS00001}attP2*	Notch-RNAi: BL33611
S5	hsFLP, FRT19A, tubGal80 / P{ry[+t7.2]=neoFRT}19A; ry[506] ; ; tub-Gal4, UAS-GFP	FRT19A: BL1709; MARCM19A: Gift of P. Jouandin
S6A, left	*w ; P{y[+t7.7] w[+mC]=GMR71F06-GAL4.C-int}attP40 / P{w[+mC]=UAS-2xEGFP}AH2 ; P{y[+t7.7] w[+mC]=Tub-GAL4.C-int}attP2*	GMR71F06-Gal4-C-int: This manuscript
S6A, middle	*w ; P{y[+t7.7] w[+mC]=Tub-GAL4.C-int}attP40 ; TI{RFP[3xP3.PB]=2A-GAL4(1-20)::N-int}Delta[G4-N-int] / P{w[+mC]=UAS-2xEGFP}AH3*	Delta-Gal4-N-int: BL602717
S6A, right	*w ; P{y[+t7.7] w[+mC]=GMR71F06-GAL4.C-int}attP40 / P{w[+mC]=UAS-2xEGFP}AH2 ; TI{RFP[3xP3.PB]=2A-GAL4(1-20)::N-int}Delta[G4-N-int]*	
S6B	*w ; P{y[+t7.7] w[+mC]=GMR71F06-GAL4.C-int}attP40 / P{w[+mC]=UAS-2xEGFP}AH2 ; TI{RFP[3xP3.PB]=2A-GAL4(1-20)::N-int}Delta[G4-N-int]*	
S6C	*w ; P{y[+t7.7] w[+mC]=GMR71F06-GAL4.C-int}attP40 / w ; P{y[+t7.7] w[+mC]=GMR71F06-GAL4.C-int}attP40 / P{w[+mC]=UAS-2xEGFP}AH2 ; TI{RFP[3xP3.PB]=2A-GAL4(1-20)::N-int}Delta[G4-N-int] ; TI{RFP[3xP3.PB]=2A-GAL4(1-20)::N-int}Delta[G4-N-int] / P{w[+mC]=tubP-GAL80[ts]}ncd[GAL80ts-7]*	GTRACE: BL28280
S7	*w ; P{UAS-Stinger}2 / + ; P{y[+t7.7] w[+mC]=GMR71F06-GAL4}attP2 / MKRS*	UAS-Stinger: BL90920,

		GMR71F06-Gal4: BL39596
S8	*w ; P{UAS-Stinger}2 / + ; P{y[+t7.7] w[+mC]=GMR71F06-GAL4}attP2 / MKRS*	UAS-Stinger: BL90920, GMR71F06-Gal4: BL39596
S9	See Figure 8.	
S10A, left	*w ; P{w[+mC]=tubP-GAL80[ts]}10], P{UAS-Stinger}2 ; P{y[+t7.7] w[+mC]=GMR71F06-GAL4}attP2 / +*	
S10A, middle	*w / y sc v sev ; P{w[+mC]=tubP-GAL80[ts]}10], P{UAS-Stinger}2 ; P{y[+t7.7] w[+mC]=GMR71F06-GAL4}attP2 / P{y[+t7.7] v[+t1.8]=VALIUM20-mCherry.RNAi}attP2*	mCherry RNAi: BL35785
S10A, right	*w ; P{w[+mC]=tubP-GAL80[ts]}10], P{UAS-Stinger}2 / fzr RNAi GD25550 ; P{y[+t7.7] w[+mC]=GMR71F06-GAL4}attP2*	fzr RNAi: VDRC GD-25550
S10B, left	*w ; P{w[+mC]=tubP-GAL80[ts]}10], P{UAS-Stinger}2 ; P{y[+t7.7] w[+mC]=GMR71F06-GAL4}attP2 / +*	
S10B, middle	*w / y sc v sev; P{w[+mC]=tubP-GAL80[ts]}10], P{UAS-Stinger}2 ; P{y[+t7.7] w[+mC]=GMR71F06-GAL4}attP2 / P{y[+t7.7] v[+t1.8]=VALIUM20-mCherry.RNAi}attP2*	mCherry RNAi: BL35785
S10B, right	*w / y sc v sev ; P{w[+mC]=tubP-GAL80[ts]}10], P{UAS-Stinger}2 / + ; P{y[+t7.7] w[+mC]=GMR71F06-GAL4}attP2 / P{y[+t7.7] v[+t1.8]=TRiP.HMS01541}attP2*	E2F1 RNAi: BL36136
S10C, left	*w ; P{w[+mC]=tubP-GAL80[ts]}10], P{w[+mC]=UAS-2xEGFP}AH2 ; P{y[+t7.7] w[+mC]=GMR71F06-GAL4}attP2 / +*	
S10C, middle	*w / y sc v sev; P{w[+mC]=tubP-GAL80[ts]}10], P{w[+mC]=UAS-2xEGFP}AH2 ; P{y[+t7.7] w[+mC]=GMR71F06-GAL4}attP2 / P{y[+t7.7] v[+t1.8]=VALIUM20-mCherry.RNAi}attP2*	mCherry RNAi: BL35785
S10C, right	*w / y y sc v sev; P{w[+mC]=tubP-GAL80[ts]}10], P{w[+mC]=UAS-2xEGFP}AH2 ; P{y[+t7.7] w[+mC]=GMR71F06-GAL4}attP2 / P{y[+t7.7] v[+t1.8]=TRiP.HMS01538}attP2*	myc RNAi: BL36123
S10D-G, left	*w ; P{w[+mC]=tubP-GAL80[ts]}10], P{w[+mC]=UAS-2xEGFP}AH2 ; P{y[+t7.7] w[+mC]=GMR71F06-GAL4}attP2 / +*	
S10D-G, middle	*w / y sc v sev ; P{w[+mC]=tubP-GAL80[ts]}10], P{w[+mC]=UAS-2xEGFP}AH2 ; P{y[+t7.7] w[+mC]=GMR71F06-GAL4}attP2 / P{y[+t7.7] v[+t1.8]=VALIUM20-mCherry.RNAi}attP2*	mCherry RNAi: BL35785
S10D-G, right	*w / y v ; P{w[+mC]=tubP-GAL80[ts]}10], P{w[+mC]=UAS-2xEGFP}AH2 ; P{y[+t7.7] w[+mC]=GMR71F06-GAL4}attP2 / P{y[+t7.7] v[+t1.8]=TRiP.JF02473}attP2*	cycE RNAi: BL29314
S11	Same as 9A	

Click or tap here to enter text.
