# Supplementary material for: The Drosophila proventriculus lacks stem cells but compensates for age-related cell loss via endoreplication-mediated cell growth
Source: Nat Commun. 2026 Jan 27;17:2086. doi: 10.1038/s41467-026-68876-5 (PMC12954101; doi:10.1038/s41467-026-68876-5)
Supplement: Supplementary file 3 — Reporting Summary [file 41467_2026_68876_MOESM3_ESM.pdf]

Corresponding author(s): Ben Ewen-Campen, Norbert Perrimon

Last updated by author(s): 01/12/2026

Reporting Summary

Nature Portfolio wishes to improve the reproducibility of the work that we publish. This form provides structure for consistency and transparency in reporting. For further information on Nature Portfolio policies, see our Editorial Policies and the Editorial Policy Checklist.

Statistics

For all statistical analyses, confirm that the following items are present in the figure legend, table legend, main text, or Methods section.

n/a	Confirmed	
☐	☒	The exact sample size (*n*) for each experimental group/condition, given as a discrete number and unit of measurement
☐	☒	A statement on whether measurements were taken from distinct samples or whether the same sample was measured repeatedly
☐	☒	The statistical test(s) used AND whether they are one- or two-sided<br>*Only common tests should be described solely by name; describe more complex techniques in the Methods section.*
☒	☐	A description of all covariates tested
☐	☒	A description of any assumptions or corrections, such as tests of normality and adjustment for multiple comparisons
☐	☒	A full description of the statistical parameters including central tendency (e.g. means) or other basic estimates (e.g. regression coefficient) AND variation (e.g. standard deviation) or associated estimates of uncertainty (e.g. confidence intervals)
☐	☒	For null hypothesis testing, the test statistic (e.g. *F*, *t*, *r*) with confidence intervals, effect sizes, degrees of freedom and *P* value noted<br>*Give P values as exact values whenever suitable.*
☒	☐	For Bayesian analysis, information on the choice of priors and Markov chain Monte Carlo settings
☒	☐	For hierarchical and complex designs, identification of the appropriate level for tests and full reporting of outcomes
☒	☐	Estimates of effect sizes (e.g. Cohen's *d*, Pearson's *r*), indicating how they were calculated

Our web collection on statistics for biologists contains articles on many of the points above.

Software and code

Policy information about availability of computer code

Data collection	*Provide a description of all commercial, open source and custom code used to collect the data in this study, specifying the version used OR state that no software was used.*
Data analysis	GraphPad Prism 10.5.0; aravis Vision 4D (Zeiss, v4.1.2); FIJI 2.1.0; Cell Ranger software (10x Genomics, v7.0.0); R (v4.3); Seurat (v4.3.0). Scripts for snRNAseq analysis: https://github.com/MujeebQadiri/SingleNucleiProventriculusAnalysis/tree/main.

For manuscripts utilizing custom algorithms or software that are central to the research but not yet described in published literature, software must be made available to editors and reviewers. We strongly encourage code deposition in a community repository (e.g. GitHub). See the Nature Portfolio guidelines for submitting code & software for further information.

Data

Policy information about availability of data

All manuscripts must include a data availability statement. This statement should provide the following information, where applicable:

- Accession codes, unique identifiers, or web links for publicly available datasets
- A description of any restrictions on data availability
- For clinical datasets or third party data, please ensure that the statement adheres to our policy

The snRNA-seq data generated in this study have been deposited in the NCBI Gene Expression Omnibus (GEO) database under accession code GSE301009 [https://www.ncbi.nlm.nih.gov/geo/query/acc.cgi?acc=GSE301009]. The snRNA-seq Atlas is available for data mining at https://www.flyrnai.org/scRNA/. The raw data used to generate all figures are provided as a Source Data file.

Research involving human participants, their data, or biological material

Policy information about studies with human participants or human data. See also policy information about sex, gender (identity/presentation), and sexual orientation and race, ethnicity and racism.

Reporting on sex and gender	N/A
Reporting on race, ethnicity, or other socially relevant groupings	N/A
Population characteristics	N/A
Recruitment	N/A
Ethics oversight	N/A

Note that full information on the approval of the study protocol must also be provided in the manuscript.

Field-specific reporting

Please select the one below that is the best fit for your research. If you are not sure, read the appropriate sections before making your selection.

[x] Life sciences [ ] Behavioural & social sciences [ ] Ecological, evolutionary & environmental sciences

For a reference copy of the document with all sections, see nature.com/documents/nr-reporting-summary-flat.pdf

Life sciences study design

All studies must disclose on these points even when the disclosure is negative.

Sample size	No statistical method was used to predetermine sample sizes. Sample sizes were determined based on pilot experiments and practical feasibility.
Data exclusions	No data were excluded from the results.
Replication	Experiments were designed to establish robust differences between genotypes and were not replicated with the following exceptions: gut leakiness assays were performed in three separate replicates; Psuedomonas infection was performed in duplicate and both replicates were consistent (a single experiment is shown in the manuscript, as pooling these two independent tests would be inappropriate.)
Randomization	Samples were allocated based on genotype and treatment, and were not randomized.
Blinding	No blinding was performed. The experiments were performed and analyzed by the same people, making blinding impractical.

Reporting for specific materials, systems and methods

We require information from authors about some types of materials, experimental systems and methods used in many studies. Here, indicate whether each material, system or method listed is relevant to your study. If you are not sure if a list item applies to your research, read the appropriate section before selecting a response.

Materials & experimental systems

n/a	Involved in the study	
[ ]	[x]	Antibodies
[x]	[ ]	Eukaryotic cell lines
[x]	[ ]	Palaeontology and archaeology
[ ]	[x]	Animals and other organisms
[x]	[ ]	Clinical data
[x]	[ ]	Dual use research of concern
[x]	[ ]	Plants

Methods

n/a	Involved in the study	
[x]	[ ]	ChIP-seq
[x]	[ ]	Flow cytometry
[x]	[ ]	MRI-based neuroimaging

Antibodies

Antibodies used	Rabbit anti-GFP AlexaFluor488 conjugate (Molecular Probes A-21311) Used at 1:300)<br>Wg (Developmental Studies Hybridoma Bank 4D4) used at 1:100<br>rabbit anti-phophohistone 3 (Cell Signaling Technologies 9701S) Used at 1:500
Validation	Rabbit anti-GFP Alexafluor 488: https://www.thermofisher.com/antibody/product/GFP-Antibody-Polyclonal/A-21311?

ef_id=Cj0KCQiAi9rJBhCYARIsALyPDttED4iMg81eNAFSYgJFPBB1vcuRabJHr-VtOP5EuLdivzS2ylan33waApSLEALw_wcB:G:s&s_kwcid=AL!3652!3!459736943987!!!g!!!10950825775!106531320406&cid=bid_pca_aup_r01_co_cp1359_pjt0000_bid00000_0se_gaw_dy_pur_con&gad_source=1&gad_campaignid=10950825775&gclid=Cj0KCQiAi9rJBhCYARIsALyPDttED4iMg81eNAFSYgJFPBB1vcuRabJHr-VtOP5EuLdivzS2ylan33waApSLEALw_wcB

Wg: https://dshb.biology.uiowa.edu/4D4

anti-pH3: https://www.cellsignal.com/products/primary-antibodies/phospho-histone-h3-ser10-antibody/9701?srsltid=AfmBOornRidVdwfoaQYPkbkKzn9gKxtMZnVM2rvfPxf5zcnBqrDF_veY

Animals and other research organisms

Policy information about studies involving animals; ARRIVE guidelines recommended for reporting animal research, and Sex and Gender in Research

Laboratory animals	Drosophila melanogaster; the specific genotypes and their ources are provided as a table in the manuscript. Age was controlled for each experiment, as described in the methods section of the manuscript.
Wild animals	No wild animals were used in this study.
Reporting on sex	All studies were performed on adult, mated female flies.
Field-collected samples	No field-collected samples were used.
Ethics oversight	No ethical approval was required for studies on Drosophila melanogaster.

Note that full information on the approval of the study protocol must also be provided in the manuscript.

Plants

Seed stocks	N/A
Novel plant genotypes	N/A
Authentication	N/A
